# Verification of Accuracy of Genomically Enhanced Predicted Transmitting Ability Techniques in Predicting Milk and Fat Production in Holstein Cattle in Taiwan

**DOI:** 10.3390/ani15223334

**Published:** 2025-11-19

**Authors:** Chun-Hsuan Chao, Jen-Wen Shiau

**Affiliations:** Northern Region Branch, Taiwan Livestock Research Institute, Ministry of Agriculture, No 207-5, Bitoumian, Miaoli County 36843, Taiwan; jwshiau@tlri.gov.tw

**Keywords:** Holstein cattle, genetic testing, genomically enhanced predicted transmitting ability

## Abstract

This study evaluated the predictive performance of genomically enhanced predicted transmitting abilities for milk (gPTAM) and fat yield (gPTAF) in 986 first-lactation Holstein cows from 25 herds in Taiwan. Ordinary least squares and linear mixed models revealed significant positive associations between genomic predictions and observed yields (milk: β = 1.201, R^2^ = 0.469; fat: β = 1.444, R^2^ = 0.507). Incorporating herd and birth-year effects improved model fit and reduced residual variability. Five-fold cross-validation confirmed the robustness of the full mixed model, with predictive R^2^ increasing to 0.293 for milk and 0.363 for fat, distinct from the OLS R^2^ (0.469 and 0.507) representing phenotypic variation explained, indicating moderate predictive ability of genomic PTA values under subtropical production conditions. Model adequacy checks supported appropriate model specification, with only a mild outlier signal in the milk model. Regional analysis revealed a significant genotype-by-environment interaction for PTAF (*p* = 0.018) but not for PTAM, indicating reduced prediction accuracy in environmentally variable regions and highlighting trait-specific environmental sensitivity. Quartile stratification by gPTA and Net Merit Score demonstrated clear yield gradients, confirming both the predictive and economic value of genomic evaluations under subtropical dairy production systems.

## Simple Summary

This study examined how well genetic information can predict milk and fat production in Holstein dairy cows in Taiwan. By analyzing records from 986 cows across 25 farms, we found that cows with higher genetic scores for milk and fat production generally produced more milk and butterfat in their first lactation. When farm and birth-year effects were considered, prediction accuracy improved substantially (R^2^ increased from 0.12 to 0.47), emphasizing that environmental and management factors strongly influence gene expression in subtropical herds. The results also showed that milk production was more stable across farms, while fat yield was more sensitive to environmental conditions such as feed and climate. These findings highlight the value of integrating genomic evaluations with farm-level breeding strategies to enhance productivity and genetic progress in subtropical dairy systems like Taiwan.

## 1. Introduction

Genomic selection based on single-nucleotide polymorphism (SNP) markers has transformed animal breeding by providing a more accurate, cost-efficient, and rapid method for estimating breeding values [1,2]. Unlike conventional methods that rely solely on pedigree and phenotypic data, genomic prediction incorporates dense marker information across the entire genome, enabling greater selection accuracy, shorter generation intervals, and accelerated genetic progress. Since the release of the first genome-wide SNP chip (BovineSNP50 BeadChip) in 2007 and the launch of genomic evaluation for U.S. Holsteins in 2009, genomic selection has become a cornerstone of modern dairy breeding programs worldwide [3,4,5].

Large-scale genomic evaluations, such as those conducted by the Council on Dairy Cattle Breeding (CDCB), now encompass more than 50 economically important traits and are applied to millions of genotyped dairy animals globally [6,7]. These systems have demonstrated the capacity of genomic information to guide selection decisions and increase breeding efficiency. However, prediction accuracy may decline when genomic evaluation models are applied to populations or production systems that differ from the reference population, particularly in subtropical regions, where climatic stressors and management practices diverge substantially from temperate systems [8,9].

Previous research has examined genomic prediction accuracy for health and fertility traits in commercial U.S. Holstein herds [10,11] and evaluated their economic value using selection indices such as genomic PTA [12]. Nevertheless, empirical validation of these genomic evaluations in subtropical production environments remains limited—especially in Taiwan, where heat stress and environmental variability are key production constraints. Subtropical conditions in Taiwan, characterized by high humidity, elevated temperature–humidity index (THI), and differences in forage quality and feeding management, can alter the expression of production traits and reduce the reliability of genomic prediction models developed in temperate regions. Moreover, G × E interactions may influence the predictive accuracy of certain traits, particularly fat yield, which is more sensitive to energy balance, feed quality, and climatic fluctuations.

Economic selection indices such as NM$ are widely implemented internationally to support balanced selection goals that integrate production, fertility, and health. However, how these economic indices translate into actual production outcomes across genomic merit classes (e.g., quartiles) remains underexplored in subtropical environments.

To address these gaps, this study leveraged data from 986 Holstein cows across 25 herds in nine regions of Taiwan to: (i) evaluate the predictive accuracy of gPTAM and gPTAF under subtropical conditions; (ii) examine regional differences and G × E interactions in genomic prediction performance; and (iii) assess economic implications through NM$ index evaluation and quartile stratification. This study represents the first comprehensive validation of U.S.-derived genomic PTA models in Taiwanese Holstein cows, providing both scientific evidence and practical breeding insights to support regionally adapted genomic selection strategies in subtropical dairy production systems.

## 2. Materials and Methods

### 2.1. Animal Selection and Sampling

This study included 986 randomly selected cows from 25 herds across Taiwan. Hair follicle and blood samples were collected from each individual using a method consistent with that described in Chao et al. [13]. The animal use protocol was approved by the Institutional Animal Care and Use Committee of the Taiwan Livestock Research Institute (LRI-IACUC108-1 and LRI-IACUC109-1). Pedigree information—including birth dates, sire and dam IDs—and biological samples were submitted to Neogen Genomics (Lincoln, NE, USA), a CDCB-certified laboratory. Genotyping was performed using GGP Bovine 100 K SNP chips based on Illumina Infinium technology, along with the Illumina Infinium XT genotyping assay platform [14]. Samples were genotyped using the GGP Bovine 50 K array containing 47,843 SNPs. After applying quality filters (call rate ≥ 98%, MAF ≥ 0.01, HWE *p* ≥ 1 × 10^−6^) and excluding non-autosomal SNPs, approximately 43,000 autosomal SNPs were retained for downstream analyses.

### 2.2. Lactation Data Processing

The phenotypic traits analyzed in this study included Milk305ME and Fat305ME from first-parity cows. Monthly test-day records were extracted from the official DHI database. Only lactation records with at least seven test-day observations and lactation lengths between 235 and 315 days were retained for analysis. For lactations shorter than 305 days, missing daily yield values were imputed using a linear regression method following the approach by Robert et al. [15]. Because all cows were in their first lactation, additional adjustments for age at calving were not required. This ensured that all records used in the analysis were adjusted to a standardized 305-day basis for both milk and fat yield.

### 2.3. Genomic Prediction and Regression Analysis

To evaluate the predictive performance of gPTAs for milk and fat yield traits, statistical analyses were performed using R software (version 4.5.0). Three complementary modeling approaches were implemented:

i. Simple linear regression model—gPTAM and gPTAF were treated as independent variables, with the corresponding 305-day mature equivalent milk (Milk305ME) and fat (Fat305ME) yields as dependent variables.
ii. Full fixed-effect model (OLS)—herd, birth year, and their interaction (Herd × BirthYear) were included as fixed effects in addition to gPTA, to account for environmental and cohort-level variability among farms.
iii. Linear mixed model (LMM)—herd was additionally modeled as a random effect to represent farms as a random sample from the broader dairy population and to estimate between-herd variance components reflecting management and environmental heterogeneity, while estimating fixed effects for gPTAM (or gPTAF) and birth year. The model structure was:
  Milk305ME or Fat305ME = Intercept + gPTA + BirthYear + (1∣Herd), where the random herd effect was assumed to follow a normal distribution (1|Herd)~N (0, σ^2^_Herd).

Restricted maximum likelihood (REML) estimation was used to partition fixed and random effects, providing an estimate of herd-level variance and standard deviation, which reflects the magnitude of environmental or management differences among herds.

This integrated framework allowed us to quantify the predictive contribution of genomic information alone, assess improvements after adjusting for environmental and temporal factors, and further evaluate the relative sensitivity of milk and fat traits to between-herd variability. Model fit was assessed using regression diagnostics and summary statistics, including the coefficient of determination (R^2^), adjusted R^2^, intercepts, slope estimates, F-statistics, and variance components from the mixed model. Multicollinearity among predictors was evaluated using generalized variance inflation factors (GVIF).

Residual assumptions were verified through visual and statistical diagnostics. Q–Q plots and Shapiro–Wilk tests confirmed approximate normality, while Breusch–Pagan tests were used to assess homoscedasticity. Model adequacy and residual distribution uniformity were further examined using DHARMa simulation-based diagnostics.

To evaluate model stability and generalizability, five-fold cross-validation was performed by partitioning the dataset into five subsets for iterative model training and testing. Prediction accuracy was summarized using predictive R^2^ (derived from validation folds), root mean squared error (RMSE), and mean absolute error (MAE). The coefficient of variation (CV) of R^2^ across folds quantified predictive stability, with lower CV indicating greater consistency. All statistical tests and model diagnostics were evaluated using a significance threshold of *p* < 0.05.

Scatter plots of observed versus predicted values were generated using the ggplot2 package, with separate panels for simple and full models. A 1:1 reference line was added to facilitate visual comparison of prediction accuracy.

Additionally, cows were stratified into quartile groups based on gPTAM, gPTAF, and Net Merit (NM$) values. One-way analysis of variance (ANOVA) was applied to test for differences in Milk305ME and Fat305ME among quartiles. When ANOVA results were significant (*p* < 0.05), Tukey’s Honestly Significant Difference (HSD) post hoc tests were used for pairwise comparisons. Distinct superscript letters in tables denote statistically significant differences among quartile means.

### 2.4. Evaluation of Regional Consistency and G × E Interaction

To examine whether the predictive performance of gPTAM and gPTAF varied across geographic regions, herd-level Pearson correlation coefficients between genomic predictions and observed phenotypes (gPTAM vs. Milk305ME; gPTAF vs. Fat305ME) were calculated and compared using one-way ANOVA. Each herd was assigned to one of nine major dairy regions in Taiwan (Changhua, Chiayi, Hualien, Miaoli, Nantou, Pingtung, Tainan, Taoyuan, and Yunlin) based primarily on administrative divisions, which also reflect general geographic and climatic contrasts across northern, central, southern, and eastern areas (e.g., temperature–humidity variation among regions). This regional classification allowed evaluation of genotype-by-environment effects and prediction consistency under distinct subtropical production conditions.

To further assess G × E interactions in genomic prediction accuracy, linear mixed models were fitted using the emmeans and emtrends packages in R. Region was included as a fixed effect, and herd was modeled as a random intercept. Simple slope analyses were performed to estimate regional slopes for gPTAM and gPTAF, and pairwise comparisons of slopes were conducted using Tukey’s method to identify significant regional contrasts.

An omnibus (joint) likelihood-ratio test was used to determine whether the regional effects on slopes were significant. The G × E interpretation was based on: (1) the significance of individual regional slopes, (2) pairwise regional contrasts, and (3) omnibus test results. This combined framework allowed evaluation of whether genomic prediction accuracy remained stable across regions (as observed for PTAM) or was influenced by regional environments (as observed for PTAF).

## 3. Results

### 3.1. Predictive Accuracy of Genomic Values for Milk and Fat Yield

**Figure 1:**
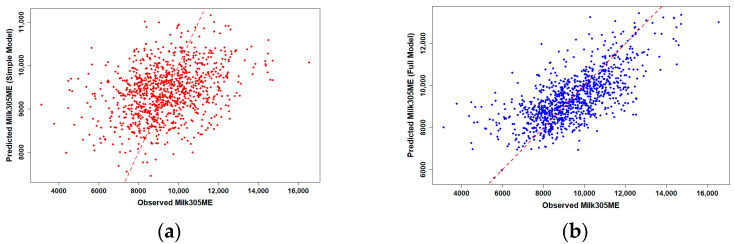
Observed versus predicted 305-day milk yield in Holstein cows using genomic PTA models (*n* = 986). (**a**) Simple linear regression using gPTAM only showed moderate predictive ability (R^2^ = 0.117, representing the proportion of phenotypic variation explained by the model). (**b**) Multiple regression including herd, birth-year, and their interaction improved prediction accuracy substantially (R^2^ = 0.469, representing the proportion of phenotypic variation explained by the OLS model rather than the predictive R^2^ from cross-validation). The red dashed line represents the 1:1 reference line, indicating perfect prediction. The closer the points are to this line, the higher the model accuracy.

**Figure 2:**
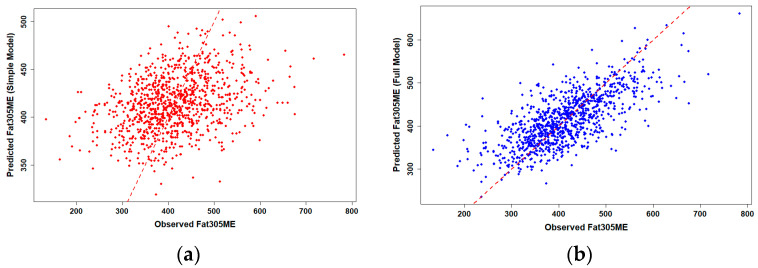
Observed versus predicted 305-day fat yield in Holstein cows using genomic PTA models (*n* = 986). (**a**) Simple linear regression using gPTAF only showed moderate predictive ability (R^2^ = 0.119, representing the proportion of phenotypic variation explained by the model). (**b**) Multiple regression including herd, birth-year, and their interaction improved prediction accuracy substantially (R^2^ = 0.507, representing the proportion of phenotypic variation explained by the OLS model rather than the predictive R^2^ from cross-validation). The red dashed line represents the 1:1 reference line, indicating perfect prediction. The closer the points are to this line, the higher the model accuracy.

To assess the predictive performance of genomic PTA values for production traits, we compared simple and full linear regression models for 305-day milk and fat yield in Holstein cows (*n* = 986). Observed versus predicted plots revealed clear differences in model performance (Figure 1 and Figure 2).

Simple linear regression using gPTAM and gPTAF alone showed only moderate predictive ability, with R^2^ values of 0.117 for milk and 0.119 for fat yield, representing the proportion of phenotypic variation explained by the simple model. When herd, birth-year, and their interaction were included in the full model, prediction accuracy increased substantially, with R^2^ values rising to 0.469 for milk and 0.507 for fat yield, corresponding to the explanatory R^2^ of the OLS model rather than the predictive R^2^ from cross-validation, suggesting that herd-level management consistency and environmental uniformity accounted for much of the observed phenotypic variation across farms. Data points in the full model clustered more tightly around the 1:1 reference line, indicating markedly improved prediction accuracy compared with the simple model.

Pearson correlation analysis further supported these results, showing moderate but consistent associations between genomic predictions and observed performance across the population (r ≈ 0.34 for both traits). This indicates that genomic PTA values provide a meaningful basis for predicting phenotypic outcomes under subtropical production conditions. Simple linear regression models quantified these effects in more detail (Table 1). For milk yield, the estimated regression coefficient was β = 1.1895 (*p* < 2 × 10^−16^) with an intercept of 9366.18, explaining 11.7% of the phenotypic variation (R^2^ = 0.117, adj. R^2^ = 0.116). For fat yield, the estimated coefficient was β = 1.3526 (*p* < 2 × 10^−16^) with an intercept of 426.03, explaining 11.9% of the variation (R^2^ = 0.119, adj. R^2^ = 0.118). These results confirm that while gPTAM and gPTAF alone provide moderate predictive power, incorporating environmental and cohort effects can markedly enhance genomic prediction accuracy.

Model-diagnostic plots for the full OLS models exhibited approximately linear Q–Q patterns with only minor tail deviations (Figures S1 and S2), indicating near-normal residual distributions. Although formal tests detected slight departures from normality, these were negligible given the large sample size. Residual–fitted plots showed no systematic trends, supporting model adequacy. Tests for heteroscedasticity confirmed homogeneous variance across predicted values, and generalized variance inflation factors (GVIF) were well below commonly accepted thresholds. Overall, these diagnostics indicated that multicollinearity, residual structure, and variance heterogeneity did not materially affect the validity of the regression estimates. Detailed test statistics are provided in Supplementary Tables S1 and S2.

To further evaluate model robustness and generalizability, five-fold cross-validation was performed for both simple and full models (Table 2). For milk yield, the cross-validated R^2^ increased from 0.117 in the simple model to 0.293 in the full model, with RMSE decreasing from 1634.3 to 1503.7 kg and MAE from 1278.2 to 1151.5 kg. The CV of R^2^ across folds was 13.2%, indicating moderately stable predictive performance with some variability across folds. For fat yield, the R^2^ increased from 0.122 to 0.363, with RMSE decreasing from 78.9 to 68.3 kg and MAE from 61.3 to 52.6 kg. However, the R^2^ CV was 23.2%, suggesting greater sensitivity to herd or environmental variability.

In addition to the fixed-effects models, LMM analysis was conducted to account for herd-level clustering effects by including herd as a random intercept and birth year as a fixed effect.

For milk yield, PTAM remained a strong and significant predictor (β = 1.201, *p* < 0.001), and herd-level variance (SD = 853.3 kg) indicated substantial differences among farms, reflecting the influence of management intensity, housing system, and feeding practices on yield variability, while birth-year effects were not significant. For fat yield, PTAF also remained highly significant (β = 1.444, *p* < 0.001), but herd-level variance was smaller (SD = 43.9 kg), suggesting that fat yield was less affected by inter-farm management heterogeneity compared with milk yield. DHARMa residual diagnostics for both LMMs indicated no over-/under-dispersion (milk: *p* = 0.92; fat: *p* = 0.854) and acceptable residual patterns. The milk model showed a small outlier signal (outlier test *p* = 0.015), whereas the fat model did not (*p* = 0.454) (Figures S3 and S4). These diagnostic checks support the adequacy of the LMM specification and the robustness of the fixed-effect estimates.

Taken together, the diagnostic results (normality, homoscedasticity, multicollinearity, and DHARMa) support the validity of inference from both OLS and LMM frameworks. The consistency between LMM and multiple regression findings reinforces the critical contribution of herd-level structure to predictive performance—particularly for milk yield. Herd-level and cohort effects account for a substantial proportion of the variation in production, and integrating these factors significantly enhances the predictive accuracy of genomic PTA models. Moreover, cross-validation analysis confirmed that the milk yield model exhibited more stable prediction across validation folds, whereas the fat yield model was more sensitive to environmental variation. These results highlight that genomic PTA values (gPTAM and gPTAF) provide robust genetic signals across herds, but the extent of herd-level variance differs between traits, reflecting their environmental sensitivity.

### 3.2. Regional Variation in the Predictive Accuracy of gPTAM and gPTAF

The predictive performance of genomic evaluations was examined across nine major dairy production regions in Taiwan (Changhua, Chiayi, Hualien, Miaoli, Nantou, Pingtung, Tainan, Taoyuan, and Yunlin). Correlation coefficients between genomic predicted transmitting abilities and actual 305-day production traits were calculated to assess the consistency of prediction accuracy across regions (Table 3).

**Figure 3:**
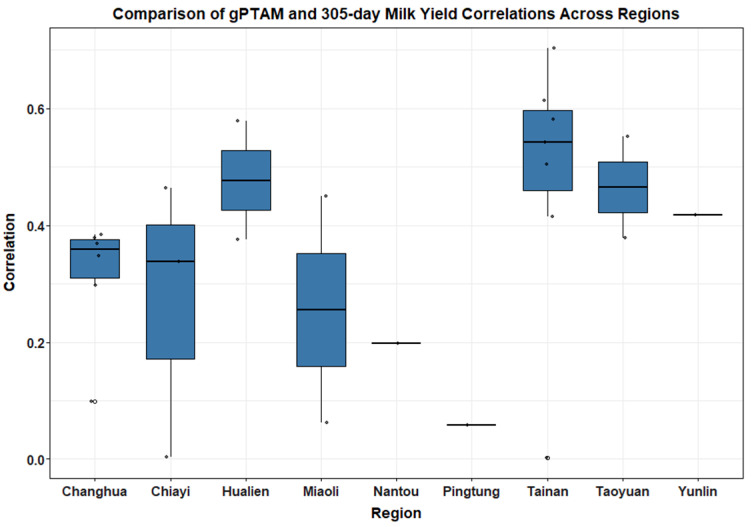
Regional variation in gPTAM–Milk305ME correlations. Boxplots show the distribution of correlation coefficients between gPTAM and Milk305ME across nine regions in Taiwan. Each box represents the interquartile range (IQR), the horizontal line indicates the median, and whiskers extend to the minimum and maximum values within 1.5 × IQR. Regional differences in predictive accuracy are visually evident, with Tainan and Taoyuan herds exhibiting higher median correlations (∼0.5), while Nantou and Pingtung show much lower values, suggesting variability in genomic prediction performance across geographical locations, likely influenced by differences in heat-stress exposure and on-farm management systems.

**Figure 4:**
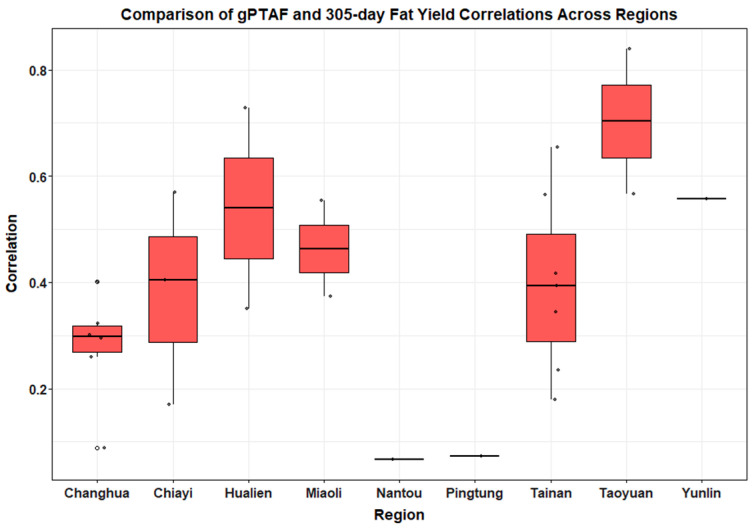
Regional variation in gPTAF–Fat305ME correlations. Boxplots depict the correlation coefficients between gPTAF and Fat305ME across nine regions in Taiwan. The median IQR, and whiskers (extending to 1.5 × IQR) are shown for each region. Taoyuan and Hualien displayed the strongest predictive correlations.

Boxplots (Figure 3 and Figure 4) illustrated clear regional patterns in genomic prediction accuracy. For gPTAM and Milk305ME, Tainan and Hualien exhibited the highest median correlation values (0.54 and 0.48, respectively), indicating stronger genomic predictive performance in these regions. In contrast, Pingtung and Nantou showed the lowest correlations (0.06 and 0.2, respectively), suggesting lower prediction reliability in these areas. These regional differences likely reflect variation in farm management intensity, feed quality, and cooling infrastructure between regions; herds in southern coastal areas such as Pingtung are more frequently exposed to high heat load and humidity, whereas inland mountain areas like Nantou experience greater diurnal temperature variation and feed supply fluctuation, both of which can reduce the stability of genomic predictions.

A similar trend was observed for gPTAF and Fat305ME, with Taoyuan showing the strongest median correlation (approximately 0.7), followed by Yunlin and Hualien. Nantou and Pingtung again exhibited lower correlation values, indicating regional disparities in the predictive accuracy for fat yield.

One-way ANOVA results indicated no significant differences in milk yield prediction across regions (F(8, 16) = 1.107, *p* = 0.408), whereas fat yield prediction showed significant variation (F(8, 16) = 2.6, *p* = 0.0494). These findings were further supported by G × E simple slopes analysis. PTAM slopes did not differ significantly among regions (χ^2^ = 6.224, *p* = 0.621) (Table 3), confirming stable genomic prediction for milk yield. In contrast, PTAF slopes varied significantly (χ^2^ = 18.496, *p* = 0.018), with Taoyuan exhibiting the strongest slope (3.85), significantly higher than several other regions (*p* < 0.05). Model checks did not indicate heteroscedasticity or lack of fit at the regional level, and LMM estimates remained consistent with OLS.

### 3.3. Genomic Quartile Analysis and Economic Interpretation

To further evaluate the practical implications of genomic values, cows were stratified into quartiles based on their gPTAM and gPTAF values, and corresponding production traits were compared. For gPTAM, the average 305-day milk yield increased progressively with genomic merit. Specifically, cows in the lowest quartile (Q1, bottom 25%) produced 8655 ± 1537 kg of milk, while those in the second (Q2), third (Q3), and top quartiles (Q4) yielded 9246 ± 1604 kg, 9463 ± 1661 kg, and 10,265 ± 1785 kg, respectively (Table 4). Similarly, cows grouped by gPTAF quartiles demonstrated ascending fat production, with Q1 yielding 381 ± 77 kg of fat, Q2 yielding 404 ± 82 kg, Q3 yielding 423 ± 81 kg, and Q4 reaching 453 ± 79 kg. Statistical analysis confirmed that the differences in both milk and fat yield among quartiles were highly significant (*p* < 0.0001), suggesting that genomic predictions have strong discriminatory power in identifying higher-yielding animals.

From an economic standpoint, the production difference between Q4 and Q1 cows in terms of milk yield amounted to 1610 kg per lactation. Assuming a representative raw-milk price of US $1.1 per kilogram for illustrative purposes, this difference translates into an estimated profit advantage of US $1771 per cow. These estimates are based on assumed milk prices and do not account for farm-specific feed-cost variation, but they highlight the potential economic relevance of selecting genetically superior animals.

Additional quartile analysis was conducted using NM$ values. Cows in the top NM$ quartile yielded 9687 ± 1496 kg of milk, compared to 9085 ± 1716 kg in the lowest quartile—a 602 kg difference, equating to a profit margin of approximately US$704 per cow (Table 5). Fat yield also increased across NM$ quartiles, from 389 ± 81 kg to 443 ± 76 kg (Table 6), underscoring the comprehensive economic benefit of using NM$ in selection decisions.

Together, these findings confirm that genomic evaluation, especially when interpreted through quartile stratification, not only predicts performance outcomes but also provides actionable economic insights to support genomic-based selection strategies in Taiwanese Holstein herds.

## 4. Discussion

### 4.1. Enhancing Genomic Prediction Performance with Environmental and Cohort Adjustments

This study provides strong evidence supporting the practical relevance of genomic evaluations in Taiwan’s Holstein dairy herds under subtropical production conditions. The significant associations between gPTAM and gPTAF with actual first-lactation milk and fat yields confirm the utility of genomic information for improving selection accuracy. Specifically, the positive regression coefficients indicate that cows with higher genomic PTA values tend to exhibit superior phenotypic performance, reaffirming the value of genomic selection as a key breeding strategy [16].

Importantly, incorporating herd, birth-year, and their interaction into the regression models markedly improved predictive performance, with R^2^ increasing from 0.117 to 0.469 for milk yield and from 0.119 to 0.507 for fat yield. Linear mixed model analysis further highlighted that herd-level variance explained a considerable proportion of total variation, particularly for milk yield, underscoring the role of herd management and production environments in shaping phenotypic expression. This finding is consistent with recent evidence showing that environmental effects and G × E interactions must be modeled to avoid underestimating true genetic potential [17].

Model robustness was further supported by five-fold cross-validation. Cross-validated R^2^ increased from 0.117 to 0.293 for milk yield and from 0.122 to 0.363 for fat yield, accompanied by reductions in RMSE and MAE. The CV of R^2^ was moderate for milk (13.2%), indicating relatively stable prediction across validation folds, but higher for fat (23.2%), suggesting greater environmental sensitivity. These results align with international studies showing that integrating genetic, environmental, and management information improves model accuracy and generalizability [18].

In addition to model fit performance, model diagnostics confirmed the adequacy of the statistical framework. GVIF values were below 1.6 for both traits, indicating low multicollinearity. DHARMa residual diagnostics for the LMMs showed no evidence of over- or under-dispersion (milk: *p* = 0.92; fat: *p* = 0.854) and acceptable residual patterns. A small outlier signal was observed in the milk model (outlier test *p* = 0.015), whereas the fat model did not show significant deviation (*p* = 0.454). These diagnostic checks support the validity of inference from both OLS and LMM frameworks and confirm the robustness of the fixed-effect estimates.

Linear mixed model analysis further clarified the contribution of herd-level effects to genomic prediction accuracy. PTAM remained a strong predictor of milk yield (β = 1.201, *p* < 0.001), with a larger herd-level variance (SD = 853.3 kg), reflecting considerable between-herd differences in environmental or management conditions. PTAF also showed a significant genetic effect (β = 1.444, *p* < 0.001) but a smaller herd-level variance (SD = 43.9 kg), suggesting that fat yield is less influenced by between-herd variability. Together, the diagnostics and validation results indicate that milk yield predictions exhibited more stable out-of-sample performance, whereas fat yield was more sensitive to environmental variation, likely due to metabolic adjustments under heat stress, where cows prioritize maintenance and thermoregulation over lipid synthesis.

### 4.2. Regional Variation and G × E Effects

A key contribution of this study is the inclusion of regional validation and G × E analyses. While no significant regional differences were detected for gPTAM (χ^2^ = 6.224, *p* = 0.621), the predictive accuracy for gPTAF varied significantly across regions (χ^2^ = 18.496, *p* = 0.0178). Notably, the Taoyuan region exhibited the steepest genetic slope (3.85), which was significantly higher than several other regions in pairwise comparisons. This pattern reflects the higher environmental sensitivity of fat yield compared with milk yield, which remained more stable across regions. These findings are consistent with reports showing that milk volume tends to be genetically robust, while fat and protein yields are more responsive to environmental conditions and management practices.

Early research demonstrated that genetic variance for milk and fat yield increased with production level, suggesting a genotype-by-environment interaction underlying yield differences among herds [19]. Later studies in Australia confirmed that fertility and production traits were influenced by regional climatic variation and herd management differences [20]. More recently, reaction norm analyses in European dual-purpose cattle also revealed that feeding and housing systems strongly contribute to G × E variability, particularly for milk yield and conformation traits [21].

Regional heterogeneity in the present study may therefore arise from differences in feed composition, barn cooling systems, climatic conditions, herd genetic connectedness, or data recording quality. Integrating regional calibration steps into genomic evaluation pipelines may further enhance predictive reliability, particularly for environmentally responsive traits such as fat yield. The significant G × E interaction detected for gPTAF (*p* = 0.018) suggests that environmental conditions modulate the expression of genetic potential for fat yield in Taiwanese Holstein populations. Regional differences, particularly the weaker predictive efficiency observed in Pingtung and Nantou, may reflect variations in microclimate, feeding management, or herd composition across farms. Comparable findings have been reported in Australia, where neglecting G × E effects has been shown to bias genetic evaluations conducted under heterogeneous environmental and management conditions [20].

This finding supports the notion that genomic prediction models should incorporate region-specific environmental covariates to improve robustness. Future research may explore the integration of climate indices, feeding systems, or management-level descriptors to better capture G × E effects in subtropical dairy systems. This study provides one of the first pieces of evidence quantifying G × E interactions in genomic evaluations of Holstein cattle under subtropical production conditions.

The observed G × E interaction for fat yield emphasizes the importance of considering environmental covariates in genomic evaluation systems. As noted by Tiezzi and Maltecca [22], accounting for G × E responses is crucial for sustainable livestock breeding under diverse climatic and management conditions.

### 4.3. Practical Applicability and Study Novelty

Beyond the identification of regional G × E effects, this study also provides partial validation evidence supporting the transferability of U.S.-based genomic evaluation systems to subtropical Holstein populations. Previous genomic studies have predominantly focused on temperate dairy systems in North America, where environmental variation is relatively moderate. However, dairy herds in Taiwan face substantial heat stress, high humidity, and variable management conditions that may challenge the transferability of genomic evaluations.

The present findings confirm that gPTA values for milk and fat yield derived from the U.S. reference population remain significantly correlated with realized production performance in Taiwanese Holstein cows, supporting the robustness and cross-population applicability of genomic predictions. This contributes novel evidence on the adaptability of genomic evaluation systems across climatic zones and management structures. Establishing a dedicated reference population for Taiwan that integrates local environmental and management data would further strengthen genomic evaluation accuracy and ensure sustainable genetic progress under subtropical dairy systems.

### 4.4. Economic Implications of Genomic Stratification

Stratifying cows into quartiles based on gPTAM, gPTAF, and NM$ revealed clear performance gradients, with top quartile animals outperforming the lowest quartile in both milk and fat yield. These results highlight the economic and practical value of applying genomic selection to sire selection and replacement heifer programs. When genomic predictions are validated regionally—especially for fat yield—breeding strategies can be optimized to match local production systems, ensuring greater genetic gains and more predictable on-farm outcomes.

### 4.5. Role of NM$ and Broader Breeding Implications

The validation of NM$ as a multi-trait selection index in this study strengthens its value as a tool for balancing production traits with fertility, health, and longevity. Combining NM$ with single-trait gPTAs provides a more comprehensive and economically meaningful basis for selection, consistent with strategies adopted in advanced dairy industries.

Taken together, these findings demonstrate that genomic PTA values form the genetic foundation of breeding decisions, while herd-level, cohort-level, and regional factors act as amplifiers or modulators of this genetic potential. Genomic selection alone is not sufficient; it must be combined with environmental, management, and diagnostic validation strategies to achieve optimal results under subtropical production conditions.

## 5. Conclusions

This study demonstrated that genomically enhanced predicted transmitting abilities (gPTAM and gPTAF) are reliable indicators of first-lactation milk and fat yield in Holstein cows under Taiwan’s subtropical production conditions. Incorporating herd and cohort effects markedly improved predictive accuracy (R^2^ = 0.469 for milk; 0.507 for fat), while five-fold cross-validation confirmed model robustness (R^2^ = 0.293 for milk; 0.363 for fat) with reduced RMSE and MAE. Linear mixed model results revealed strong genetic effects for PTAM (β = 1.201) and PTAF (β = 1.444), and greater herd-level variance for milk (SD = 853.3 kg), indicating stronger environmental influence on milk yield.

Model diagnostics, including multicollinearity (GVIF < 1.6) and DHARMa residual tests, confirmed adequate model fit, with only a minor outlier signal in the milk model. A significant genotype-by-environment interaction for PTAF (*p* = 0.018) suggested higher environmental sensitivity for fat yield. Overall, these findings highlight that genomic PTA values provide a robust genetic foundation, while herd and regional factors modulate their phenotypic expression. Future genomic evaluations should incorporate environmental covariates such as temperature–humidity index and feed system descriptors to improve prediction accuracy and facilitate the selection of heat-resilient Holstein genotypes in subtropical dairy systems. Integrating genomic selection with regionally adapted management strategies can enhance prediction reliability and accelerate sustainable genetic improvement in subtropical dairy systems.

## Figures and Tables

**Table 1:** Results of simple linear regression models predicting 305-day milk and fat yields from genomic PTA values in Holstein cows.

Dependent Variable	Predictor (gPTA)	β (Estimate)	SE	t-Value	*p*-Value	R^2^	n
Milk305ME (kg)	PTAM (kg)	1.1895	0.1041	11.43	<0.001	0.1172	986
Fat305ME (kg)	PTAF (kg)	1.3526	0.1174	11.52	<0.001	0.1189	986

**Table 2:** Cross-validation results for simple and full models predicting 305-day milk and fat yields in Holstein cows.

Trait	Model	R^2^ (CV)	RMSE (kg)	MAE (kg)	CV of R^2^ (%)
Milk305ME	Simple	0.117	1634.3	1278.2	–
Milk305ME	Full	0.293	1503.7	1151.5	13.2
Fat305ME	Simple	0.122	78.9	61.3	–
Fat305ME	Full	0.363	68.3	52.6	23.2

**Table 3:** The coefficients of the correlations of the gPTAM and gPTAF values with the first lactation milk yield and fat yield in tested herds.

Herd	Region	No. of Genotyped Cattle	Correlation Between gPTAM and Milk305ME	Correlation Between gPTAF and Fat305ME
1	Changhua	247	0.30	0.26
2	Chiayi	150	0.46	0.57
3	Miaoli	115	0.45	0.55
4	Changhua	78	0.38	0.30
5	Hualien	65	0.38	0.35
6	Tainan	32	0.61	0.18
7	Taoyuan	26	0.38	0.57
8	Yunlin	25	0.42	0.56
9	Tainan	24	0.42	0.39
10	Tainan	22	0.70	0.34
11	Tainan	21	0.00	0.56
12	Changhua	21	0.38	0.30
13	Tainan	20	0.58	0.42
14	Changhua	20	0.35	0.09
15	Changhua	17	0.10	0.40
16	Hualien	15	0.58	0.73
17	Nantou	13	0.20	0.07
18	Tainan	12	0.54	0.65
19	Tainan	11	0.50	0.23
20	Pingtung	10	0.06	0.07
21	Miaoli	10	0.06	0.37
22	Taoyuan	9	0.55	0.84
23	Changhua	9	0.37	0.32
24	Chiayi	8	0.00	0.17
25	Chiayi	6	0.34	0.40
Total		986		

**Table 4:** Estimated simple slopes of gPTAM and gPTAF on actual 305-day milk and fat yield (Milk305ME and Fat305ME) across nine geographic regions in Taiwan. Slopes represent the strength of the association between genomic predicted transmitting abilities and observed phenotypes within each region. Omnibus tests indicate whether regional differences in slopes are statistically significant. PTAM slopes did not differ among regions (*p* = 0.621), whereas PTAF slopes showed significant variation (*p* = 0.018), with Taoyuan exhibiting the strongest genetic effect.

Region	PTAM Slope	SE (PTAM)	*p*-Value (PTAM)	PTAF Slope	SE (PTAF)	*p*-Value (PTAF)
Changhua	1.105	0.162	<0.0001	1.046	0.189	<0.0001
Chiayi	1.251	0.242	<0.0001	1.422	0.269	<0.0001
Hualien	1.346	0.295	<0.0001	1.676	0.351	<0.0001
Miaoli	0.931	0.255	0.0003	1.167	0.317	0.0002
Nantou	0.720	0.816	0.3779	0.007	1.207	0.9951
Pingtung	0.215	1.159	0.8530	0.352	1.226	0.7739
Tainan	1.556	0.241	<0.0001	1.647	0.331	<0.0001
Taoyuan	1.791	0.543	0.0010	3.851	0.741	<0.0001
Yunlin	1.276	0.517	0.0136	1.713	0.594	0.0039
Omnibus test	–	–	χ^2^ = 6.224, *p* = 0.621	–	–	χ^2^ = 18.496, *p* = 0.018

**Table 5:** Average first milk yield levels of four gPTAM and NM$-based quartiles (genetic groups) (*p* < 0.05).

Item	Genetic Group	Milk Yield (kg)
PTAM	Worst 25	8665 ± 1537 ^c^
	26–50%	9211 ± 1597 ^ab^
	51–76%	9432 ± 1643 ᵇ
	Best 25	10263 ± 1787 ^a^
NM$	Worst 25	9088 ± 1741 ᵇ
	26–50%	9388 ± 1853 ^ab^
	51–76%	9399 ± 1793 ^ab^
	Best 25	9703 ± 1945 ^a^

^abc^ Marginal means within column and milk yield with different superscripts differ (*p* < 0.05).

**Table 6:** Average first fat yield levels of four gPTAF and NM$-based quartiles (genetic groups).

Item	Genetic Group	Fat Yield (kg)
PTAF	Worst 25	380.8 ± 76.9 ^d^
	26–50%	403.7 ± 81.8 ^c^
	51–76%	422.4 ± 81.3 ^b^
	Best 25	452.9 ± 79.3 ^a^
NM$	Worst 25	388.6 ± 82.1 ^c^
	26–50%	405.5 ± 83.5 ^bc^
	51–76%	419.6 ± 84.3 ^b^
	Best 25	443.5 ± 76.6 ^a^

^a–d^ Marginal means within column and milk yield with different superscripts differ (*p* < 0.05).

## Data Availability

The data presented in this study are available on request from the corresponding author. The data are not publicly available due to institutional data protection policies and agreements with participating farms.

## Supplementary Materials

The following supporting information can be downloaded at: https://www.mdpi.com/article/10.3390/ani15223334/s1, Figure S1: Model-diagnostic plots for the full OLS model of milk yield.; Figure S2: Model-diagnostic plots for the full ordinary least squares (OLS) model of fat yield.; Figure S3: DHARMa residual diagnostic plots for the LMM of milk yield.; Figure S4: DHARMa residual diagnostic plots for the LMM of fat yield.; Table S1. Residual diagnostics for milk and fat yield models using Shapiro–Wilk and Breusch–Pagan tests.; Table S2: Multicollinearity diagnostics for milk and fat yield models using GVIF.

## Abbreviations

The following abbreviations are used in this manuscript:

gPTAM	Genomic Predicted Transmitting Ability for Milk yield
gPTAF	Genomic Predicted Transmitting Ability for Fat yield
PTA	Predicted Transmitting Ability
OLS	Ordinary Least Squares
LMM	Linear Mixed Model
GVIF	Generalized Variance Inflation Factor
RMSE	Root Mean Squared Error
MAE	Mean Absolute Error
CV	Coefficient of Variation
NM$	Net Merit Dollar
G × E	Genotype-by-Environment Interaction
DHI	Dairy Herd Improvement
SD	Standard Deviation
