# Supplementary material for: Verification of Accuracy of Genomically Enhanced Predicted Transmitting Ability Techniques in Predicting Milk and Fat Production in Holstein Cattle in Taiwan
Source: Animals (Basel). 2025 Nov 19;15(22):3334. doi: 10.3390/ani15223334 (PMC12649444; doi:10.3390/ani15223334)
Supplement: Supplementary file 1 [file animals-15-03334-s001.zip › animals-3989895-supplementary.pdf]

Supplementary information

Table S1. Residual diagnostics for milk and fat yield models using Shapiro–Wilk and Breusch–Pagan tests.

Model	Test	Statistic	df	p-value
Milk	Shapiro–Wilk	W = 0.9876	—	2.90×10^{-7}
Milk	Breusch–Pagan	BP = 145.69	120	0.055
Fat	Shapiro–Wilk	W = 0.9926	—	9.91×10^{-5}
Fat	Breusch–Pagan	BP = 140.71	120	0.095

Note. Shapiro–Wilk tests indicated small deviations from normality, which are expected in large samples; however, residual–fitted plots did not reveal systematic structure. Breusch–Pagan tests were non-significant for both models ($p > 0.05$), supporting the assumption of homoscedasticity.

Table S2. Multicollinearity diagnostics for milk and fat yield models using GVIF.

Model	Predictor	GVIF	Df	GVIF^(1/(2*Df))
Milk	PTAM	1.32	1	1.15
	NM	1.65	1	1.29
	Herd	4.10	24	1.03
	BirthYear	4.15	9	1.08
Fat	PTAF	2.34	1	1.53
	NM	2.53	1	1.59
	Herd	4.35	24	1.03
	BirthYear	4.15	9	1.08

GVIF values for categorical predictors (Herd and BirthYear) are elevated due to multiple levels, but adjusted GVIF^(1/(2*Df)) values remain below the commonly accepted threshold of 2, indicating no multicollinearity concerns.

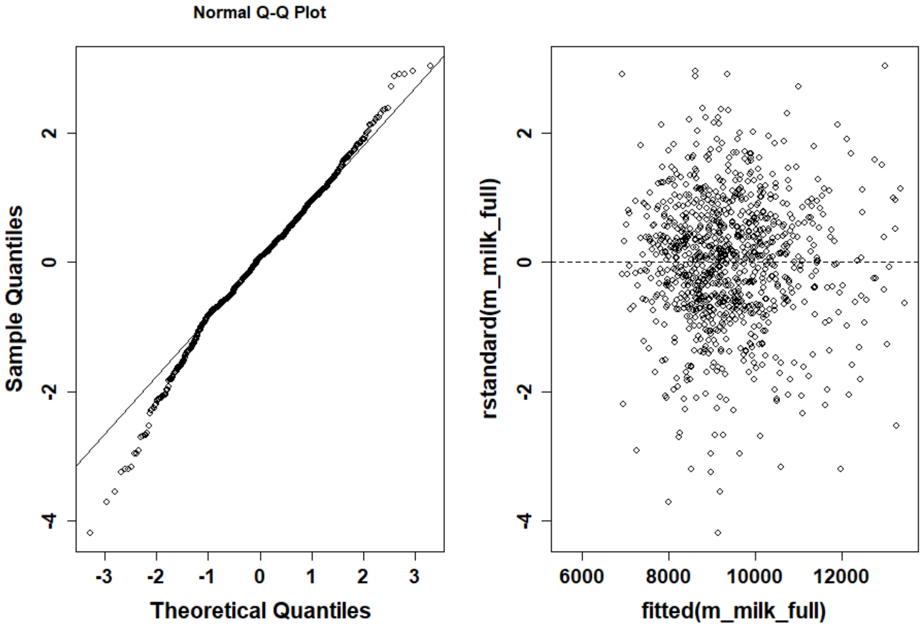

Figure S1. Model-diagnostic plots for the full OLS model of milk yield. The Q–Q plot (left) indicates approximately linear residual distribution with minor deviations at the tails, suggesting near-normality. The residuals-versus-fitted plot (right) shows random scatter around zero without systematic structure, confirming homoscedasticity and adequate linear model fit.

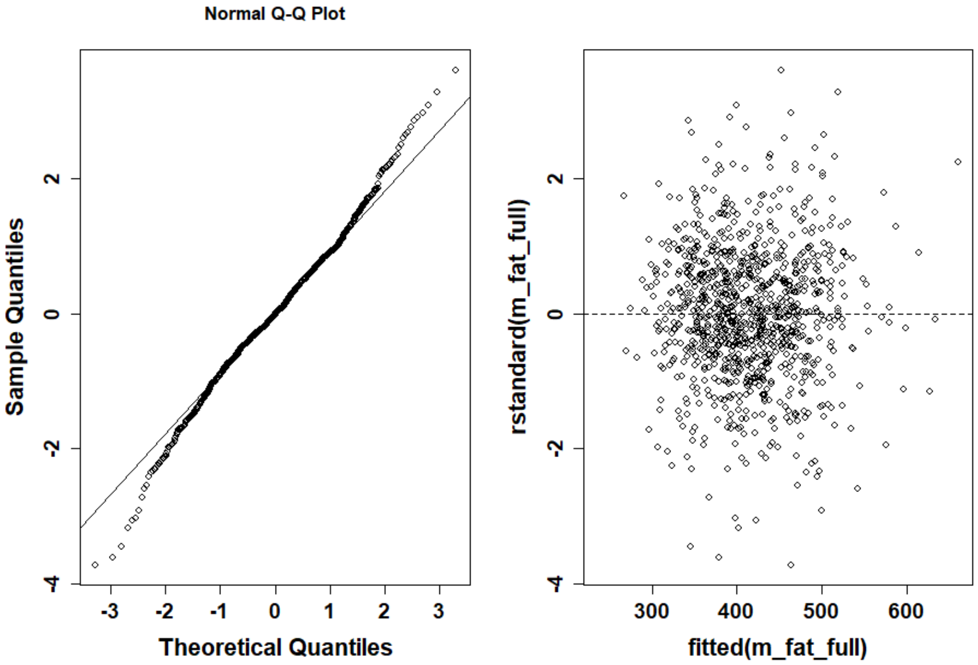

Figure S2. Model-diagnostic plots for the full ordinary least squares (OLS) model of fat yield.

The Q–Q plot (left) shows an approximately linear residual distribution with slight deviations at the extreme tails, indicating near-normality. The residuals-versus-fitted plot (right) displays random scatter around zero without systematic patterns, suggesting homoscedasticity and an adequate linear model fit.

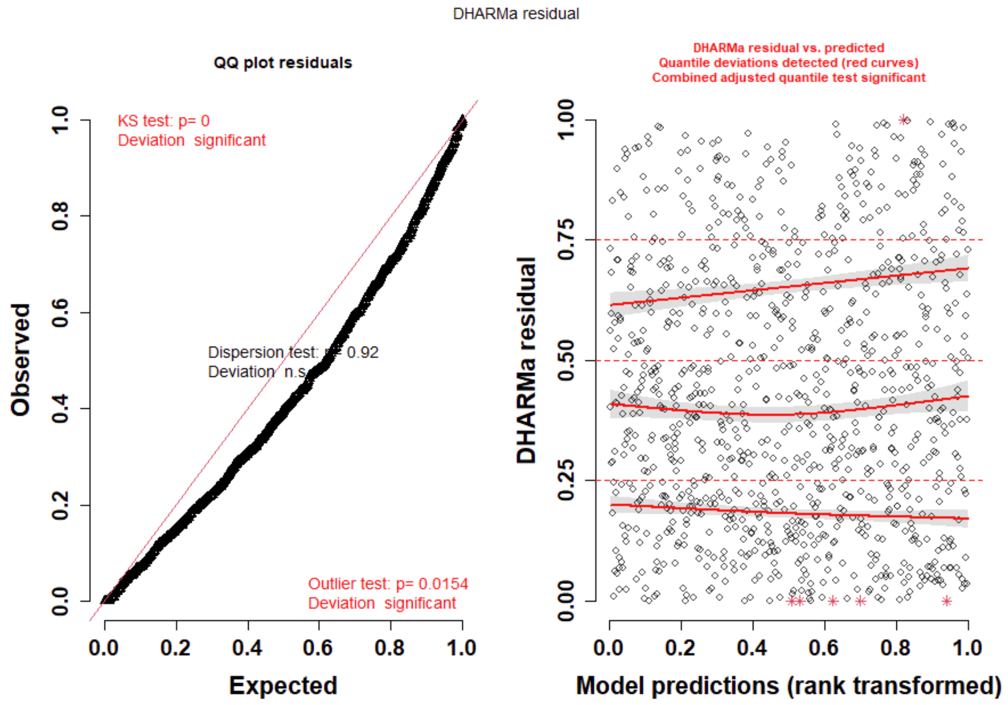

Figure S3. DHARMa residual diagnostic plots for the LMM of milk yield. The Q–Q plot of simulated residuals (left) shows no evidence of over- or under-dispersion (dispersion test p = 0.92) and generally well-distributed residuals, though a small outlier signal was detected (outlier test p = 0.015). The residuals-versus-predicted plot (right) displays minor deviations in the quantile lines but no systematic bias, indicating an overall adequate model fit.

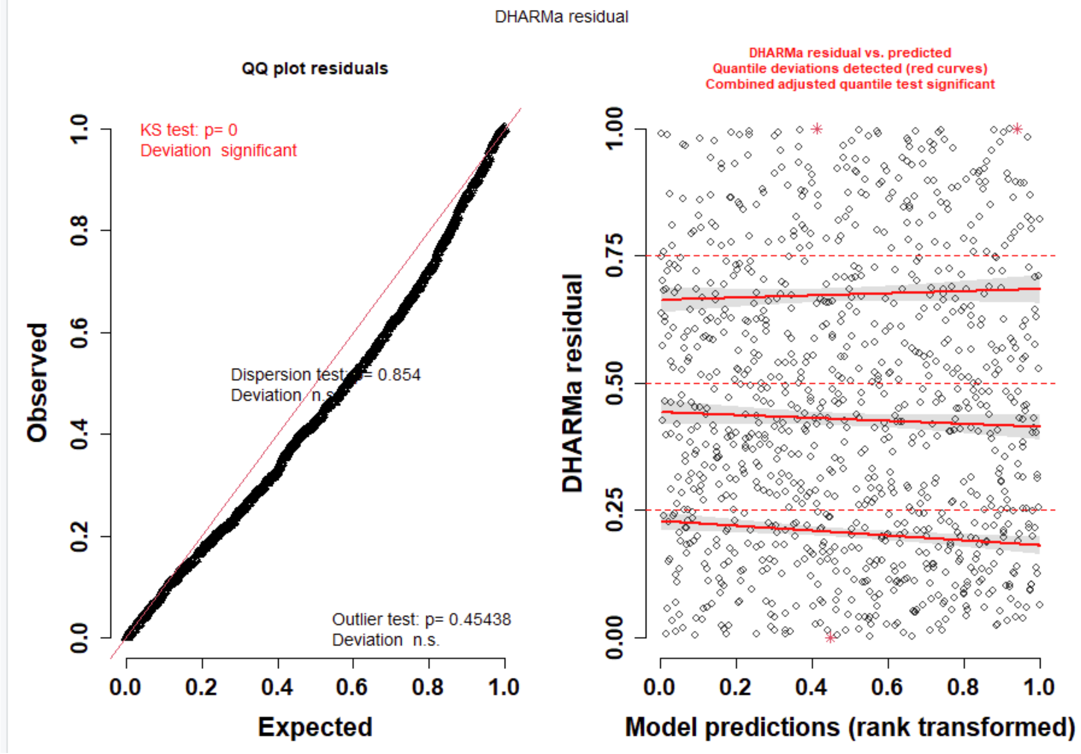

Figure S4. DHARMa residual diagnostic plots for the LMM of fat yield. The Q–Q plot of simulated residuals (left) shows no indication of over- or under-dispersion (dispersion test p = 0.854) and a near-uniform distribution without outlier signals (outlier test p = 0.454). The residuals-versus-predicted plot (right) displays no systematic pattern, confirming homoscedasticity and an adequate model fit for the LMM of fat yield.
